# Children with Autism Understand Indirect Speech Acts: Evidence from a Semi-Structured Act-Out Task

**DOI:** 10.1371/journal.pone.0142191

**Published:** 2015-11-09

**Authors:** Mikhail Kissine, Julie Cano-Chervel, Sophie Carlier, Philippe De Brabanter, Lesley Ducenne, Marie-Charlotte Pairon, Nicolas Deconinck, Véronique Delvenne, Jacqueline Leybaert

**Affiliations:** 1 Université libre de Bruxelles, Brussels, Belgium; 2 Centre de Ressource Autisme de l’Université libre de Bruxelles-‘Autrement’, Hôpital Universitaire des Enfants Reine Fabiola, Brussels, Belgium; University of Dundee, UNITED KINGDOM

## Abstract

Children with Autism Spectrum Disorder are often said to present a global pragmatic impairment. However, there is some observational evidence that context-based comprehension of indirect requests may be preserved in autism. In order to provide experimental confirmation to this hypothesis, indirect speech act comprehension was tested in a group of 15 children with autism between 7 and 12 years and a group of 20 typically developing children between 2:7 and 3:6 years. The aim of the study was to determine whether children with autism can display genuinely contextual understanding of indirect requests. The experiment consisted of a three-pronged semi-structured task involving Mr Potato Head. In the first phase a declarative sentence was uttered by one adult as an instruction to put a garment on a Mr Potato Head toy; in the second the same sentence was uttered as a comment on a picture by another speaker; in the third phase the same sentence was uttered as a comment on a picture by the first speaker. Children with autism complied with the indirect request in the first phase and demonstrated the capacity to inhibit the directive interpretation in phases 2 and 3. TD children had some difficulty in understanding the indirect instruction in phase 1. These results call for a more nuanced view of pragmatic dysfunction in autism.

## Introduction

Specific difficulties in pragmatic dimensions of language indisputably constitute one of the most salient and readily quoted correlates of Autism Spectrum Disorder (ASD). In a conversation, an addressee must possess certain pragmatic skills if he is to use context in order to reach the literal interpretation intended by the speaker. For instance, when the speaker produces an indirect request by using the interrogative sentence *Can you close the window*? correct interpretation requires that the addressee understand that the speaker is not enquiring about his physical capacity to close the window, but actually telling him to do so. By contrast, in most contexts the correct interpretation of an interrogative like *Can you run for 1 hour*? amounts to understanding it as a question. (Note that *Can you close the window*? meant as a request to close the window is not a instance of non-literal language use; compare with the same kind of indirect request meant ironically *Can you speak a bit louder*? *I’m not sure all the neighbours heard you*; cf. [1].) This paper reassesses the widespread claim that comprehension of indirect requests in ASD is impaired [2,3].

Psychologically oriented reinterpretations of philosophical work by Paul Grice [4] sparked a major revolution in the cognitive sciences, resulting in hugely popular theories that hold that all pragmatic processing involves the attribution of complex communicative intentions to the speaker; e.g. [5,6,7]. Notwithstanding significant individual differences, related to mental and verbal age, children and adults with ASD have notorious difficulties with false-belief tasks; this has led to the widespread consensus that ASD is associated with a dysfunctional Theory of Mind; e.g. [8,9,10]. The combination of this ‘mind-blindness’ theory of autism with the post-Gricean view of pragmatics as rooted within Theory of Mind naturally gave rise to a widespread expectation that every aspect of utterance interpretation that relies on contextual factors should be impaired in ASD. According to this classic view on pragmatics in autism one should expect indirect request comprehension to be deficient in children with ASD.

Yet, even though pragmatic impairment in ASD has been widely researched for the last twenty years, a proper delineation of pragmatic difficulties is still lacking. There is a broad consensus that individuals with ASD struggle with ‘social’ or ‘inter-subjective’ dimensions of language use; for instance, they often fail to produce informative, new and relevant conversational contributions, and to respond on topic; e.g. [11,12–16]. However, in recent years, a monolithic view of pragmatic impairment in autism has been mitigated by the finding that metaphor interpretation [17–19] and scalar implicature derivation [20,21] are preserved, or, at least, not specifically impaired in ASD. These findings gel well with approaches to communicative disorders that conceive of pragmatics as a suite of intermeshed but distinct skills, emerging from a set of independent capacities [22]. On these views, which reject the notion of a single pragmatic module, some context-dependent aspects of utterance interpretation may rely on egocentric, salience-based heuristics, which do not necessarily involve mind-reading; e.g. [23,24]. Thus, it is perfectly reasonable to expect that such ‘egocentric’ pragmatic processing may remain intact in autism; see [25,26].

Indirect speech acts raise a number of issues both in theoretical and experimental pragmatics; e.g. [27,28,29,26,30]. However, the following rough definition, which should suffice here, is uncontroversial enough: A speaker performs an indirect speech act when the linguistic structure of her utterance does not provide sufficient cues to understand, out of context, which speech act—assertion, comment, request, advice, order etc.—the speaker is performing. Consider the examples in (1) and (2); the interrogative sentence in (1) will often be used to ask a question about the addressee’s age; likewise, the declarative in (2) will often be uttered as a comment on a toy.

1. How old are you?
2. This is a nice toy.

These communicative functions are closely tied with these examples’ linguistic structure: one needs virtually no context to understand (1) as a question and (2) as a comment. For this reason, we may say that, under the readings just provided, (1) is a *direct* question and (2) is a *direct* comment. However, (1) may also be used as a request, directed, for instance, at a child who misbehaves and (2) as a suggestion to choose a particular toy. Interpreted in this way, (1) and (2) are *indirect* speech acts; quite some contextual work is needed to reach these indirectly conveyed meanings. Furthermore, it is reasonable to expect, at least prima facie, that this interpretation process requires forming some hypotheses about what the speaker intended to mean by her utterance.

As already mentioned, according to a traditional view of pragmatics in autism, one should expect individuals with ASD to experience difficulty in grasping indirect speech acts. As it happens, earlier studies have yielded mixed results in this respect. Paul and Cohen’s [3] participants with autism performed well on conventionalised or quite simple indirect requests of the form *Can you colour this circle blue*?*; Why not colour this circle blue*?*; You should colour this circle blue; I’ll be happy if you colour this circle blue*, but had problems with more complex sentences, such as *I’ll be sad unless you colour this circle blue* or *Doesn’t it need to be coloured blue*?. It should be noted, however, that these are rather convoluted means to perform a request, without any obvious relevance in the experimental setting. In fact, Paul and Cohen’s stimuli were borrowed from a study by Clark and Lucy [31], which showed that neuro-typical adults also have trouble understanding such complex indirect requests. Ozonoff and Miller [32] used pre-recorded stories, some of which ended with an interrogative sentence. Depending on the previous context, this sentence was to be interpreted as (a direct) question or as an indirect request. Participants were then asked to choose an ending consistent with the story they just heard. Whereas participants with ASD chose fewer correct endings in general than controls, they chose significantly fewer endings compatible with the ‘direct question’ meaning than those compatible with the indirect ‘request meaning’. MacKay and Shaw [2] report that children with high-functioning autism or Asperger syndrome have trouble explaining why a protagonist used an indirect request. An obvious limitation in this study is that such a meta-communicative task provides only an indirect window on actual processing, which may be obscured by the additional complexities of the task.

A much more categorical finding emerged from an observational study of eleven French-speaking children with low-functioning autism, filmed in a familiar setting while interacting with familiar adults by Kissine, De Brabanter and Leybaert [33]. Measuring compliance with requests, they found that all requests were complied with well above chance level. More importantly, requests constituted by sentences with an unambiguous interrogative intonation (3), a declarative sentence (4) or a verbless sub-sentential fragment (5) were complied with as much as those cast in the imperative mood (6).

1. Tu fermes le pot? (= You close the jar?)
2. Tu vas remettre la bouteille dans ton cartable (= You are going to put the bottle back in you bag)
3. Ta place (= Your seat; [meaning: get back to your seat])
4. Verse le lait (= Pour the milk)

While sentences in the imperative mood, such as (6), are prototypically used for performing requests [34], the linguistic structure of the interrogative in (3), of the declarative in (4) or of the noun phrase in (5) does not suffice to understand that they are meant as a request. Consequently, some kind of contextual processing is required to comprehend (and then to comply with) the request. What Kissine et al.’s [33] results show, then, is that children with autism do not rely exclusively on the linguistic meaning. Since it is quite unlikely that children in their study engaged in sophisticated reasoning about the speaker’s communicative intentions, their compliance with non-imperative requests probably reveals some kind of egocentric pragmatic processing. It makes perfect sense to assume that indirect requests can very often be grasped by considering the interpretation that is the most salient from one’s own point of view, without drawing any inferences about what the speaker intended to convey.

However, in the corpus analysed by Kissine et al. [33] requests usually concerned some salient action, and it is possible that children used the utterance or some of its components as a cue towards this action. Of course, such a pragmatic strategy would still amount to combining linguistic meaning and the surrounding context to reach the communicated meaning. However, while one can conclude from Kissine et al.’s data that children with autism are somehow *guided* by contextual factors towards a motor response, it remains to be seen whether they can use context to *choose* a particular interpretation. In other words, in order to demonstrate that children with autism display genuine understanding of indirect requests, one needs to show that they can understand a certain target-sentence as an indirect request in one context and that they avoid this interpretation in a context where the same target-sentence is meant as a direct comment or a direct question.

The present paper constitutes an experimental sequel to Kissine et al. [33]. In line with the results of this study, our first hypothesis is that children with autism will understand indirect requests. Based on this first hypothesis, we expect children to comply with instructions framed in an indirect way, such as *He has no hat* meant as a request to put a hat on a doll. Furthermore, in line with the idea of preserved egocentric pragmatic processing in autism [25,26], our second hypothesis is that this comprehension will not result from an automatic association of a cue word with an action; in other words, we expect that children with autism will display awareness of the context of interaction. A good way to test this hypothesis is to see whether children with autism can inhibit the ‘request’, directive interpretation of a sentence, which was previously used as an indirect request, in a context where this interpretation is not appropriate; e.g. they should not interpret *He has no hat* as a request in a context where it is manifestly used as a comment.

Before proceeding to a description of our method for testing these hypotheses, it is worth raising two methodological issues. First, it is increasingly accepted that research on language development should privilege ecological act-out paradigms over metalinguistic or elicitation tasks whose intrinsic difficulty may obliterate good performance on the linguistic skill under investigation e.g. [35]. This is all the more true for children with ASD, who may be distressed by an unfamiliar situation and feel puzzled or challenged by a naming task or pointing tasks. Therefore, in order to test indirect speech act comprehension, one has to build an experimental setting where the two interpretations (direct and indirect) of the target-sentence are as natural as possible; moreover, the correct interpretation should be elicited as a behaviour pattern that is fully natural for the child and embedded within an engaging activity.

The second methodological point concerns the comparison group. In experimental investigations of communicative and cognitive development in ASD it is customary to match participants with ASD by verbal and/or non-verbal age with typically developing (TD) children and/or with children with a non-autistic impairment (classically Down syndrome). This methodological choice is fully justified in studies that attempt to uncover a pattern of impairment specific to ASD. However, the hypothesis made in this paper is precisely that children with ASD may perform well on indirect speech act understanding. To emphasise, our hypothesis is not that children with ASD should display an inferior comprehension of indirect requests than TD comparison children, but, on the contrary, that their comprehension should not be specifically impaired.

Furthermore, one should not rule out a priori that such good performance is impossible without adult-level mindreading. Therefore, the ideal comparison group here should be one where good indirect speech act comprehension combines with minimal Theory of Mind; if our expectations are correct, participants with ASD should perform *at least as well as* members of such a comparison group. Of course, it is difficult to find a population with no Theory of Mind at all and sufficiently developed linguistic skills. An acceptable option for a comparison group is to recruit TD children between 2:5 and 3:5 years. Since the late seventies, it has been established that children under 3:8 years have no trouble understanding indirect speech acts [36–39]; at the same time, this age range lies below the 3 years 8 months threshold, when it is usually assumed that first-order Theory of Mind starts to be functional at an adult level. That said, several studies report signs of implicit understanding of others’ mental states around the age of two [40–42]. This kind of implicit mindreading is likely to be recruited in pragmatic processing; importantly, it has not been evidenced in ASD [43]. Therefore, if, in line with our expectations, participants with ASD and TD comparison children display comparable comprehension of indirect speech acts, one should not rush to the conclusion that this result provides evidence for identical pragmatic processing in both groups. Where young TD children can already rely on some basic Theory of Mind, children with autism may be using an alternative, ‘mindblind’ strategy.

## Method

### Population

This research has received the approval of the Ethics Committee of the Hôpital des Enfants Reine Fabiola; each participant's parents filled in an informed consent form. Sixteen French-speaking children (5 girls and 11 boys) diagnosed with autism were recruited (*a*) through the Centre de Référence Autisme at the *Hôpital Universitaire des Enfants Reine Fabiola*, Brussels, (*b*) at the school *Nos Pilifs*, specialised in the education of children with ASD, and (*c*) the *Service Universitaire Spécialisé pour personne avec Autisme*, Mons. (One girl with ASD was eventually excluded from the analyses, see below.) All children had received a formal diagnosis of autism in conformity to the criteria of *DSM-IV* (and, for 9 of them with the *ADI-R*) by a team of professional neuropsychologists, paediatricians and speech therapists specialised in ASD diagnosis. The chronological age of children with autism ranged from 7 to 12 years. Their non-verbal IQ, as measured by the *Leiter-R* ranged from 67 to 109. (For some children, the non-verbal IQ was tested using the original edition of the *Leiter* scale, and their scores were converted to match the norms of the revised edition.) The main reason for choosing this chronological age span is that language development is very often delayed in autism. Accordingly, there was a risk that younger children with autism would not have reached sufficient morpho-syntactic competence for testing indirect speech act comprehension to be tested.

Twenty-four French-speaking TD children (12 girls and 12 boys), aged between 2,7 and 3,7 years were recruited in a pre-school class in the south of Belgium; four children were eventually excluded from analyses (see below). Informed consent forms were signed by parents of all participants.

To ensure that participants with autism did not have a verbal disadvantage relative to the comparison group, every child’s receptive vocabulary was tested using the EXALANG 3–6 [44]. The EXALANG 3–6 is a computer-mediated French receptive vocabulary test, suited for children between 3 and 6 years. (The computer-mediated interface makes it particularly suitable for children with autism.) Among the 20 TD children whose results were included within the analyses three were slightly below the age of 3 (2.7, 2.8 and 2.9). Since the main aim of collecting vocabulary measures was to ensure that ASD children were not disadvantaged relative to the TD group we decided to collect EXALANG scores for these children too.

### Experimental procedure

The experiment took place in a quiet room that contained one low table with two low chairs and an office desk with another chair. Experimenter 1 (EXP1) sat with the child at the low table, on which were placed four Mr & Mrs Potato Head dolls, consisting of a head fitted with feet, and 86 parts which could be attached to the doll (arms, noses, eyes, ear-rings, glasses, hair, hats…). These elements included five hats and five pairs of glasses. Experimenter 2 (EXP2) sat at the desk, with her back to the child and to EXP1, and was ostensibly reading a magazine. The whole experiment was filmed by two video cameras, placed on two sides of the room. Each child was previously familiarised with both experimenters. During this familiarisation phase the child’s knowledge of the words to be used in the target-sentence of the experimenter was informally tested. (It emerged that all the words were known to all the children.)

#### Pre-test phase

During the pre-test phase, EXP1 explained to the child how to ‘dress’ a first Mr Potato Head, and then prompted the child to attach certain parts, using spontaneously generated utterances (e.g. *Put an arm on him*; *Don’t you want to give him a nose*? etc.). The aim of the pre-test phase was, first, to involve the child in the activity and, second, to make the directive interpretation of EXP1’s utterances salient.

#### Phase 1

Phase 1 started once the first Mr Potato Head was half-completed but still had room for adding several parts, among which either a hat or glasses. EXP1 uttered the *target-sentence*, as a prompt to pick up a hat or glasses for Mr Potato Head:

**Target-sentence:** *Oh*! *Il n’a pas de chapeau/de lunettes* [= Oh! He has no hat/glasses on!]

This target-sentence has the typical structure of a negative declarative sentence in French, and, furthermore, does not mention the action the child is supposed to perform. However, in the context at hand, it is clearly meant as a request by EXP1. The objective of *phase 1* is, then, to determine whether the participant can grasp this indirect directive meaning of a declarative sentence in a context where it is rendered salient.

#### Phase 2

While a correct response in *phase 1* clearly indicates some contextual processing, it may just be due to a bias to act on a cue occurring in the sentence, for instance, a key word like *hat*. The goal of *phase 2* is to ensure that correct responses in *phase 1* are not due to a tendency to act on such cues. *Phase 2* started with EXP1 suggesting that the child assemble a second Mr Potato Head. Once the activity was initiated, EXP2 uttered the *target-sentence*, ostensibly looking at the magazine she was reading. This second utterance of the target-sentence used exactly the same wording as in Phase 1. While, this time, the most salient interpretation of the *target-sentence* was that it was meant as a comment—on whatever picture EXP2 could be assumed to be looking at in her magazine—the child could still interpret it as a request to fit a hat/glasses on the second Mr Potato Head. The objective of *phase 2* was to determine whether the child can disengage from the ‘request’ interpretation of the target-sentence which was salient in *phase 1* and which still made sense given the on-going activity. In other words, if the child correctly interpreted the target-sentence as a request in *phase 1*, the ‘correct’ reaction in *phase 2* was not to reach for a hat/glasses.

A minor bias resulted from the choice of the target-sentence. If, in *phase 1*, the child spontaneously put the hat on Mr Potato Head before EXP1 uttered the target-sentence, EXP1 had to use the sentence *Oh*! *He has no glasses on*. Then, in *phase 2*, to avoid making the ‘request’ interpretation utterly implausible EXP2 had to wait until the child had put ears or/and a nose on Mr Potato Head; otherwise the glasses simply could not be attached to the doll. In other words, the choice of *hat* or *glasses* in the target sentence was contingent on the child’s behaviour. We trust that our results are robust enough to safely put aside this difference, which is due to the semi-structured nature of our task.

It is worth emphasising, from the outset, that lack of reaction in phase 2 in itself does not indicate anything about the interpretation of the target-sentence; rather, it is a measure for testing whether *correct* interpretation in *phase 1* was due to a bias towards acting on the basis of a key word like *hat*.

#### Phase 3

Correct response in *phases 1* and *2* constitutes a strong indication of a genuinely context-driven interpretation of the target-sentence. It can be objected, however, that absence of any response to the target sentence does not reflect a genuine contextual interpretative process, but simply lack of awareness of a speaker who is external to the immediate frame of interaction. In *phase 3* we assessed the possibility that the contrast between the absence of reaction the target-sentence in *phase 2* and to the compliance with the indirect request in *phase 1*, is due to the fact that the child is not aware of EXP2. As a reaction to EXP2’s utterance in *phase 2*, EXP1 stood up, walked next to EXP2, looked at the magazine and said *“Oh*! *That’s true*. *He has no hat/glasses on”*. The target-sentence was clearly meant, again, as a comment, but it was uttered by EXP1, who had just been interacting with the child and who previously uttered this same sentence as a request. For a child who interpreted the target-sentence as a request in *phase 1*, not reaching for a hat/glasses in *phase 3* was a strong indication that the interpretation in *phase 1* was really context-driven. (Again, in itself, absence of reaction in *phase 3* does not tell anything; it merely served as an additional assessment of the *correct* performance in *phase 1*).

#### Scoring

In *phase 1* the maximum score of 2 was assigned when the child complied with the indirect request, viz. if she put a hat/a pair of glasses on Mr Potato Head; an intermediate score of 1 was assigned when the child made a clear gesture towards reaching for a hat or a pair of glasses, but changed her mind; finally, lack of compliance was coded as 0. In *phases 2* and *3*, putting a hat/a pair of glasses on Mr Potato Head was scored 0 (as it reflected a mistaken interpretation of the target-sentence as a request, and not as a comment); a half-completed gesture towards a hat/a pair of glasses received the intermediate score of 1; absence of any gesture towards a hat/a pair of glasses received the maximum score of 2.

An important issue concerning the interpretation of participants’ behaviour is whether they actually attend to the experimenters’ utterances, which, to a certain extent, may be revealed by the child’s looking or not at the speaker. That said, the amount of gaze is not an entirely reliable indicator of attention when it comes to children with autism, who are known to allocate less visual attention to their conversational partners than TD children; e.g. [45]. With these reservations in mind, we nevertheless measured whether the child looked at EXP2 in *phase 2* and at EXP1 in *phase 3*; scoring absence of gaze as 0, a short glance as 1 and gazing towards the speaker as 2. The scoring procedure is summarised in Table 1. All the videotapes were coded independently by two judges (trained MA students in speech therapy); in case of conflicting scoring, the first author acted as a third judge. Conflict arose for 3 scores in the ASD group and 7 in the TD group, among which 3 concerned TD children who were eventually excluded from the analyses. In other words, the final proportion of disagreement was thus of 3/75 codes (0,04) for the ASD group and of 3/72 codes (0,041) for the TD group.

**Table 1:** Scoring procedure.

	*Phase 1*	*Phase 2*	*Phase 3*
Child puts the object mentioned in the target-sentence (e.g. a hat) on Mr Potato Head.	2	0	0
Half-completed gesture towards the object mentioned in the target-sentence (e.g. a hat).	1	1	1
Child does not reach for the object mentioned in the target-sentence (e.g. a hat).	0	2	2
Child looks at the speaker		2	2
Short glance at the speaker		1	1
Absence of gaze at the speaker		0	0

## Results

One girl with autism was excluded from the analyses because she continuously sought help from the experimenters at each stage of the experiment, which rendered her performance impossible to score. This left a group of 15 children with ASD (4 girls and 11 boys). Their mean age was 8.7 years, with a mean non-verbal IQ of 88.5, as measured by the Leiter-R scale. Four TD children were excluded from analyses either because they did not understand the game or because of an error in the experimental setting, leaving a comparison group of 20 TD children (11 girls and 9 boys) with a mean chronological age of 3.3 years.

EXALANG scores were collected for all participants, except one child with ASD whose EXALANG score was unavailable. The maximum score being 36, the mean score in the autism group was 35.4 (*sd* = 1), whereas the mean score in the TD group was 29.5 (*sd* = 6.02). Recall that the objective of collecting EXALANG scores was to ensure that participants with ASD had no linguistic disadvantage relative to the comparison group. The results show that our participants with autism had no such linguistic disadvantage; in fact, their scores on EXALANG were significantly superior (Welch’s t-test, *t*(20.3) = 4.3, p<0.001). Subsequent measures showed no influence of vocabulary on the main task results, and we chose not to report them here. (That said, the absence of perfect matching on verbal ability between the ASD and the TD group is a methodological weakness of our study.) Descriptive data is displayed in Table 2.

**Table 2:** Descriptive data.

	*ASD n = 15 (11 boys; 4 girls)*	*TDn = 20 (11 girls; 9 boys)*
*Chronological age* (years)	8.7 (1.89)	3.3 (0.3)
*EXALANG score*	35.4 (9.94) *	29.5 (6)
*Leiter-R*	88.5 (13.12)	

Mean, (standard deviation)

* *for the ASD group*, *n = 14 (10 boys; 4 girls)*

**Fig 1:**
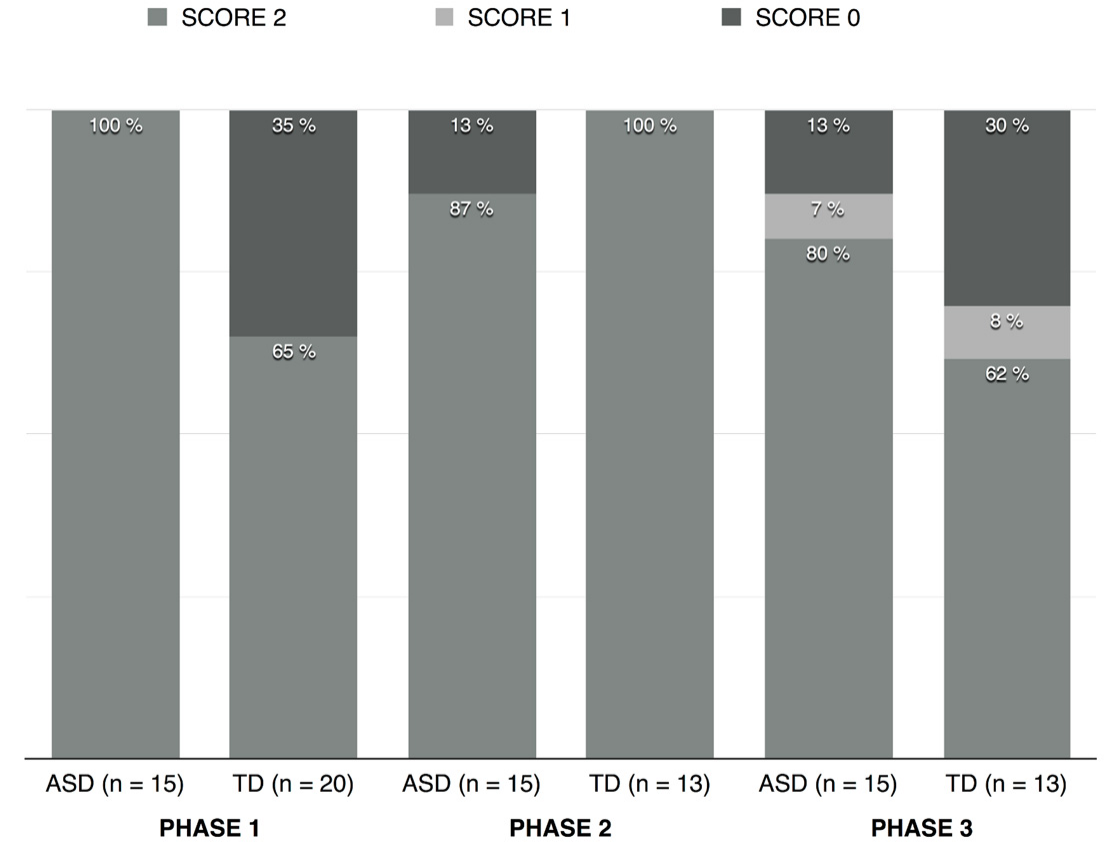
Distribution of scores per group and phase.

The distribution of scores in the main task, per group and phase, is displayed in Fig 1. As explained above, any result in *phases 2* and *3* is interpretable only if the child succeeded in *phase 1*. For this reason, TD children who did not pass *phase 1* were left out, leaving a comparison group of 13 for the analysis of *phases 2* and 3.

The first striking observation is that children with autism all reached the maximum score of 2 in *phase 1*. In other words, they all complied with the target-sentence meant as a request. Furthermore, in phase 1 TD children scored 2 significantly less often than ASD children (Mann-Whitney test, U = 97.5; p = 0.012). This result may be due to the young age of TD children. Consistent with this interpretation is the fact that no correlation between age and performance was found across the study, except for TD children in *phase 1* (Spearman’s ρ = 0.465; p< 0.05). The proportion of children whose response on *phases 2* and *3* was scored 2 is really high for both groups, and there was no significant difference between groups for *phase 2* (Mann-Whitney test, U = 84.5; p = 0.18) and for *phase 3* (Mann-Whitney test, U = 78.5; p = 0.269).

It appears, then, that in both groups children who complied with the indirect request seemed to be able to inhibit this interpretation, be it with a different (*phase 2*) or the same speaker (*phase 3*). There was no score difference between phases 1, 2 and 3 for children with autism (Friedman’s test, χr^2^ = 4.667; p > 0.05). This result confirms our hypothesis, viz. that children with autism display accurate understanding of the target-sentence across contexts of use—as a request in *phase* 1 and as a comment in *phases 2* and *3*.

However, there was a significant score difference between phases 2 and 3 for those TD children who passed *phase 1* (Friedman’s test, χr^2^ = 10; p < 0.05); in *phase 3*, TD children were more likely to misinterpret the target-sentence as a request instead of interpreting it as a comment (that is, when the target-sentence was uttered by EXP1). In fact, the task was probably more difficult for younger TD children.

**Fig 2:**
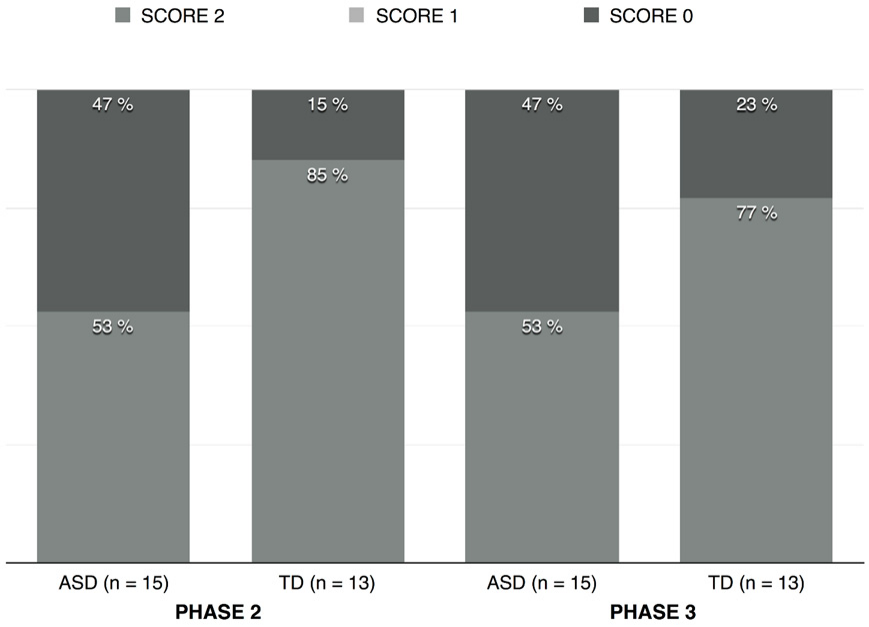
Distribution of scores for gaze per group and phase.

Fig 2 displays the distribution of scores for gaze directed at the speaker in *phases 2* and *3*, by group. While children with autism tended to look less at the speaker than TD children, this difference did not reach significance for *phase 2* (Mann-Whitney test, U = 67; p = 0.083) and for *phase 3* (Mann-Whitney test, U = 74.5; p = 0.202). It does not seem possible to conclude, then, that children with autism attended less to what EXP2 said in *phase 2* and to what EXP1 said in *phase 3*. Furthermore, in the autism group, there was no correlation between the amount of gaze and the score in *phase 2* (Spearman’s ρ = 0.419; p = 0.12) and in *phase 3* (Spearman’s ρ = 0.244; p = 0.382), which is not surprising given the high amount of maximum scores they obtained). All TD children who passed *phase* 1, hence who were included in the analysis of *phase 2*, obtained the maximal score in *phase 2* (thus rendering any correlation measure impossible). However, in *phase 3*, no correlation was observed between the score TD children obtained and the amount of gaze directed at EXP1 (Spearman’s ρ = 0.368; p = 0.216).

## Discussion

The results clearly show that children with autism can comply with an indirect request uttered in a declarative sentence form (*He has no hat*). Quite unexpectedly, in *phase 1* children with autism evidenced better understanding of indirect requests than TD children. That is, somehow in contradiction with earlier studies cf. [36,37,38], young TD children seem to find it somewhat difficult to comply with such indirect requests. One possibility is that passing our *phase 1* requires mind-reading capacities that are not sufficiently mature yet in the younger TD children of our comparison group. Note, however, that compliance is a particularly severe measure of understanding, since one may understand an utterance as a request, but, for some reason, be unwilling to comply with it. It is possible, then, that younger TD children are simply less willing to let an adult interfere with their play. In any event, it will be important at a later stage to conduct the same experiment with slightly older TD children, matched on verbal age with the ASD group.

An even more striking result is that children with autism are also capable of inhibiting the directive interpretation of the target-sentence (which was used as a request in *phase 1*) when it is uttered as a comment by the same or a different speaker. In *phase 2* when EXP2 uttered the target-sentence (e.g. *He has no hat on*), which they previously interpreted as a request, children with autism did not seem to stick to this indirect meaning. It may be the case that they simply ignored any utterance by EXP2, an adult they were not involved with during the activity. However, in *phase 3* it was EXP1, the adult with whom the child was interacting, who uttered the target-sentence as a comment; and, again, children with autism did not seem to interpret EXP1’s utterance as a request. Now, it is possible that children with autism simply ignored any utterance that is performed outside the immediate context of the Mr Potato Head activity they were involved in. First, it should be noted that some children with autism looked at the speaker in *phase 2* and in *phase 3*, which suggests that they attended to the utterance. Second, and more importantly, at the very least absence of reaction in *phases 2* and *3* indicates that children with autism are capable of identifying an interactional frame, a context of conversation, and to understand, from non-verbal cues, which utterances are external to that frame. Finally, absence of reaction to the target-sentence in phases 2 and 3 is a very strong indication that compliance with the indirect request in *phase 1* does not result from an automatized reaction to a key word or from a bias towards directive interpretations.

It can be concluded, then, that children with autism understand indirect requests relying on certain contextual cues. While our results provide an experimental confirmation to the observations by Kissine et al. [33], they do stand in contradiction with some of the earlier literature. However, as noted in the Introduction, previous studies that seemed to indicate lack of comprehension of indirect speech acts in autism either relied on quite unnatural stimuli [3] or used a meta-linguistic task [2]. Our results thus constitute one more incentive to design more ecological act-out experimental paradigms to reach better understanding of communicative difficulties in ASD.

Some readers may perhaps regret that our study does not include any measures of the children’s Theory of Mind. As things stand today, it is unclear which kind of test would be relevant here, as classic false-belief tasks involve notorious linguistic and executive confounds [25,46,47]. Furthermore, one interpretation of our results might precisely be that children with ASD display some form of understanding of the speaker’s intentions. Whether or not one endorses this explanation, the fact that children with autism display good understanding of genuinely indirect speech acts raises new questions about the nature of pragmatic processing. An issue left unresolved here is whether children with ASD and those TD children who passed our task used identical interpretative strategies. It is still unclear whether pragmatic skills underlying indirect speech act interpretation require adopting the speaker’s perspective or whether they rely solely on egocentric heuristics.
